# The Quest toward limb regeneration: a regenerative engineering approach

**DOI:** 10.1093/rb/rbw002

**Published:** 2016-03-05

**Authors:** Cato T. Laurencin, Lakshmi S. Nair

**Affiliations:** ^1^Department of Orthopaedic Surgery, Institute for Regenerative Engineering, Raymond and Beverly Sackler Center for Biomedical, Biological, Physical and Engineering Sciences, University of Connecticut Health, Farmington, CT 06030, USA,; ^2^Department of Biomedical Engineering, University of Connecticut, Storrs, CT 06269, USA,; ^3^Department of Materials Science and Engineering, University of Connecticut, Storrs, CT 06269, USA,; ^4^Department of Chemical and Biomolecular Engineering, University of Connecticut, Storrs, CT 06269, USA,; ^5^Department of Craniofacial Sciences, School of Dental Medicine, University of Connecticut Health, Farmington, CT 06030, USA.

**Keywords:** regenerative engineering, limb regeneration, stem cells, advanced biomaterial science, clinical translation

## Abstract

The Holy Grail to address the clinical grand challenge of human limb loss is to develop innovative strategies to regrow the amputated limb. The remarkable advances in the scientific understanding of regeneration, stem cell science, material science and engineering, physics and novel surgical approaches in the past few decades have provided a regenerative tool box to face this grand challenge and address the limitations of human wound healing. Here we discuss the convergence approach put forward by the field of Regenerative Engineering to use the regenerative tool box to design and develop novel translational strategies to limb regeneration.

## Introduction

Regeneration of complex tissues and organ systems such as a knee or whole limb has remained a clinical fascination in the 20th century. However, this has now been recognized as one of the Grand Challenges that need to be addressed to reach the next level of medical advances required for the 21st century [1]. It has been estimated that in the USA alone, ∼185, 000 people undergo upper or lower limb amputation every year [2]. Moreover, it has been projected that the number of people living in the USA with limb loss due to diabetes alone, can triple by 2050 [2]. Historically, prosthetic devices are used to rehabilitate those who are living with limb loss. In spite of the significant advances in improving the functions of these electromechanical devices in the past few decades, they still lack the ability to perform complex functions such as sensation for tactile input, normal gait and movement feedback [3, 4]. Surgical repair and reconstruction of human digits and limb injuries have also been attempted with some success [5, 6].

The ideal clinical strategy to address the issue of limb loss will be to regenerate a whole functional limb. The regenerative capabilities of vertebrates vary significantly from amphibians to mammals. Urodele amphibians exhibit the remarkable ability to completely regrow severed limb any time during their life time via ‘Epimorphic Regeneration’ [7, 8]. On the other hand, the amputated limbs in humans do not possess the ability to regenerate. Some studies, however, have reported the ability of digit tips to regenerate demonstrating that adult human beings still retain some regenerative potential [5, 6]. This is further corroborated by the fact that tissues such as blood and epithelia are continuously regenerated in the adult body. Bone is another example, which retains regenerative capability to a certain extent upon injury. In addition, multipotent reserve stem/progenitor cell populations have been identified through out the human body to assist the regenerative process [9]. In spite of these positive features, majority of adult human tissue fail to follow a regenerative cascade upon injury and instead follow a repair process leading to scar tissue formation [10]. The unique regenerative healing exhibited by urodele amphibians has therefore became a field of intense study to understand the characteristics of a regenerative limb [11–13].

Epimorphic regeneration occurs through the formation of a blastema, which consists of a population of progenitor cells with intrinsic morphogenetic cues capable of unfolding a highly orchestrated biological process to control the growth and pattern of the regenerating structure [7]. Briefly, upon amputation, the surface of the stump will be rapidly covered with epidermal cells to form the wound epidermis, which in turn thickens to form the apical epithelial cap (AEC). The AEC coordinates the key cellular processes involved in the regeneration. In addition, a class of proteases called matrix metalloproteinases (MMPs) plays a crucial role at this stage in extracellular matrix (ECM) remodeling, release and support the proliferation of cells from the stump tissues and present growth factors to modulate cellular activity. Under the AEC, a cluster of heterogeneous population of dedifferentiated cells aggregate to form a blastema [14, 15]. The blastema presents a highly complex gene profile due to the heterogeneity of the cell population and the multifaceted processes involved in the course of regeneration. The unique ECM of the blastema also presents directional cues to modulate cellular functions. Even though the key signaling pathways involved in blastema formation and subsequent regenerative processes are not well understood, studies have shown that some of the pathways that control embryonic limb formation such as fibroblast growth factor (FGF) and wnt-β catenin signaling are essential [16, 17]. Also, similar to embryonic development, retinoic acid and Shh signaling plays a key role in patterning and morphogenesis during limb regeneration [18, 19]. This implies that soluble factors such as retinoic acid has the potential to control the positional identity of cells and thereby impact patterns of tissue regrowth. The role of immune system (particularly macrophages) and innervation, in establishing a regenerative permissive environment in the blastema is also well recognized [20]. Even though these new insights are very encouraging, considering the diverse regenerative capabilities of the vertebrates, it is clear that only some regenerative mechanisms are conserved throughout evolution. Nevertheless, the lessons learned from the regenerative process in urodele amphibians have the potential to inform us on what is required for a regenerative limb to regenerate as well as provide hints to design novel translational strategies to induce a non-regenerative limb such as human limb to regenerate.

From an engineering perspective, the past two decades have seen significant efforts towards regenerating single tissues *in vitro* and *in vivo* using the tissue engineering approach. Tissue engineering has been defined as the application of biological, chemical and engineering principles toward the repair, restoration and regeneration of tissues using biomaterials, cells and factors alone or in combination [21]. Even though the approach led to several proof-of-concept technologies to regenerate single targeted tissues such as skin, bone and cartilage, the number of clinically available tissue engineered products are relatively low [22].

## Regenerative enginering

The limitations of the current biological and engineering approaches towards limb regeneration show that a paradigm shift is required to successfully address this Grand Challenge. A trans-disciplinary approach utilizing the cutting edge technologies currently available in disparate fields such as biology, material sciences, physical, chemical and engineering sciences, new understanding to harness the body’s innate regenerative capabilities along with early clinician participation to develop technologies that have clinical potential may hold the key to realize the dream of human limb regeneration. The research efforts in this direction led to the emergence of a new field called ‘Regenerative Engineering’ which has been defined as the Convergence of Advanced Materials Sciences, Stem Cell Sciences, Physics, Developmental Biology and Clinical Translation for the regeneration of complex tissues and organ systems [23–25]. The convergence of the trans-disciplinary cutting edge science and technologies presents exciting opportunities to seek new solutions to address the current challenges to human limb regeneration.

**Figure 1.**
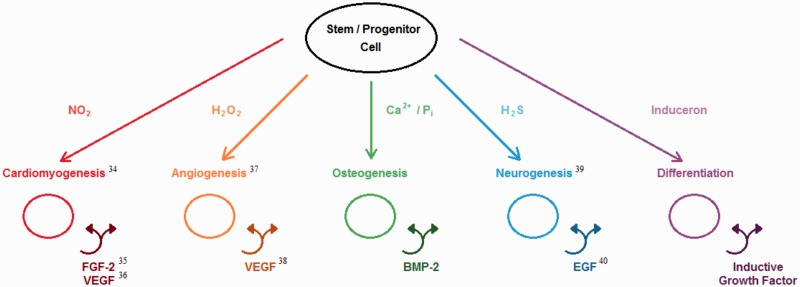
Inducerons are capable of inducing stem and progenitor cells to undergo desired differentiation and to produce their own endogenous growth factors

For instance, the outstanding research in regenerative medicine over the past few decades gave us a deeper understanding of adult and embryonic stem cells and to appreciate some similarities these cells have to blastema cells. The research also led to novel protocols to create induced pluripotent stem cells (iPSCs) from differentiated cells via the expression of few additional genes [26]. This has some stark similarities to the process of cellular dedifferentiation happening in blastema. Moreover, a recent study showed that three of the crucial iPSC genes (Sox2, Klf4 and *c*-myc) are up-regulated in regenerating newt lens and limb [27]. Considering the blastema cells being molecularly similar to a cell that has been driven to a more undifferentiated state, the possibility of controlling the extent of cellular dedifferentiation may have significant impact on developing a translational protocol for limb regeneration in humans. Some of these key innovative findings in developmental and cell biology are highly promising and can greatly inform us to address the less optimal regenerative capabilities in humans. Similarly, in the past two decades, the field of biomaterials science has significantly advanced from the level of biodegradable polymers and ceramics to custom-designed biomimetic and regenerative biomaterials. The focus in the field has been shifted from space-filling scaffolds to developing advanced biomaterials wherein the physical, mechanical and biological properties of the biomaterial scaffold can be fine tuned to enhance the natural regenerative process of the body. Studies have shown that the bioactivity of the advanced biomaterials can be significantly enhanced using biological proteins/peptides as well as biologically active effector molecules and inducerons (small molecule inducers of cell differentiation). The inducerons have the potential to make a paradigm shift in the field of regenerative engineering where they can be utilized in place of recombinant growth factors [28]. We have previously demonstrated the ability of calcium and phosphate ions as simple signaling molecules to impart intrinsic osteoinductivity by the induction of osteoinductive growth factors such as bone morphogenetic protein-2 (BMP-2) in cells [28]. Similar to calcium and phosphate ions, a large number of simple signaling molecules or inducerons are being identified (Fig. 1) which are capable of inducing stem and progenitor cell differentiation to specific lineages via autocrine and paracrine loops. Further research in these new class of bioactive molecules called ‘inducerons’ is warrantied, and they have the potential to serve as more cost-effective and safe bioactive molecules to develop advanced biomaterials. Not only the composition of the biomaterial but also the three-dimensional (3D) structure of the material has been shown to modulate cellular functions and therefore optimizing the biomaterial scaffold has the potential to create a permissive microenvironment to support regeneration. The developments in micro- and nanotechnologies lend new methodologies to create 3D biomimetic scaffolds. Last decade saw significant growth in the fabrication and characterization of nanofibrous 3D structures as biomimetic scaffolds [29]. Additive manufacturing or 3D printing is the latest innovation in this direction that has the potential to print patient-specific complex 3D organ mimetic structures to promote regeneration [30]. The role of physical forces such as electrical and mechanical forces in morphogenesis and patterning is also becoming very obvious based on some of the recent studies [31].

Using the top-down and bottom-up approaches, the field of Regenerative engineering aims to address the grand challenges in limb regeneration. The bottom-up approach is largely a cellular approach that capitalizes on the tremendous advances we are witnessing in the field of cell/molecular and developmental biology. The top-down approach aims to integrate the innovative strides in advanced material science and engineering, with cells having unique capability for morphogenesis and patterning, with physical forces that could play subtle but crucial role in morphogenesis, along with ways to modulate the immune system and enhance innervation, to recreate a highly permissive regenerative microenvironment. In summary, the convergence approach put forward by the field of regenerative engineering has the potential to bring together the tremendous innovations occurring in these distinct fields in a holistic manner to move beyond individual tissue repair to the regeneration of complex tissues and organ systems.
